# Factors associated with severity of accidental hypothermia: A cohort retrospective multi-institutional study

**DOI:** 10.1016/j.amsu.2020.04.018

**Published:** 2020-05-06

**Authors:** Patrizio Petrone, Corrado P. Marini, Ivan Miller, Collin E.M. Brathwaite, Raelina S. Howell, Dennis Cochrane, Wilson Rodríguez-Velandia, Candela Rahn, John R. Allegra

**Affiliations:** a Department of Surgery, NYU Langone Health - NYU Winthrop Hospital, NYU Long Island School of Medicine; Mineola, New York, USA; b Department of Surgery, Jacobi Medical Center; Albert Einstein College of Medicine; Bronx, New York, USA; c Department of Emergency Medicine, Westchester Medical Center; Valhalla, New York, USA; d Department of Emergency Medicine, Morristown Medical Center; Morristown, New Jersey, USA

**Keywords:** Hypothermia, Accidental, Severity

## Abstract

**Background:**

Frequently it is difficult to determine illness severity in hypothermic patients. Our goal was to determine if there are factors associated with illness severity of hypothermic emergency department (ED) patients.

**Methods:**

Multi-hospital retrospective cohort. Consecutive patients in 24 EDs (1-1-2012 to 4-30–2015). Hypothermic patients (≤35 °C) were identified using ICD codes. We used hospital admission as marker of illness severity. Student's t-test was used for differences between mean age and temperature for admitted and discharged patients. We calculated the percent of patients admitted by factor, the difference from overall admission rate and 95% confidence interval (CI) of difference.

**Results:**

There were 2094 visits with hypothermia ICD code. Of these, 132 patients had initial rectal temperatures ≤35 °C. Females comprised 42%; the mean age was 55 ± 23 years, and overall admission rate was 62%. The percent of patients with alcohol, trauma and found indoors were 39%, 27% and 27%, respectively. For admitted and discharged patients the mean ages were 60 and 48 years, respectively (p = 0.01), and initial mean temperature 32.3 °C vs. 33 °C, respectively (p = 0.07). Found indoors was associated with an 86% admission rate, a 22% increase (95% CI, 3%–34%) compared to overall admission rate. There was no statistically significant difference in admission rates from overall admission rate based on gender, alcohol or trauma.

**Conclusions:**

For hypothermic ED patients increased severity of illness was associated with older age and found indoors but not associated with initial temperature, gender, alcohol or trauma. These findings may assist physicians in treatment and disposition decisions.

## Highlights

• Accidental hypothermia is defined as a decrease in core body temperature to less than 35°C (95°F).
• Secondary hypothermia can occur in healthy and ill persons, even in warm environments, as a result of predisposing factors.
• Maintenance of a normal core temperature is achieved from a balance between heat production and heat loss.
• Factors associated with increased severity of illness for hypothermic ED patients were older age and found indoors.

## 1 Background

Accidental hypothermia (AH), defined as a decrease in core body temperature to less than 35 °C (95 °F) is a condition associated with significant morbidity and mortality [[1], [2], [3]]. While primary hypothermia occurs in healthy human beings whose ability to generate heat by means of active movement and involuntary shivering is overcome by the excessive cold in outdoor environments. Secondary hypothermia can occur in healthy as well as ill persons, even in warm environments, as a result of predisposing factors such as alcohol or drugs abuse, trauma or secondary to a variety of medical conditions leading to impaired thermoregulation through central failure (stroke, subarachnoid hemorrhage, Parkinson's disease, traumatic brain injury), endocrinologic failure (hypoadrenalism, hypopituitarism, hypoglycemia), peripheral failure (spinal cord injury, neuropathy, decreased heat production, malnutrition), and impaired heat production from a variety of causes [4].

Maintenance of a normal core temperature is achieved from a balance between heat production and heat loss. More than 90% of heat escapes through the skin by mechanisms such as radiation (55%), evaporation (25%), conduction (15%), and, to a lesser degree, convection [5]. The remaining 5% is lost via the lungs by evaporation. The most common cause of AH in an otherwise healthy, young individual is alcohol intoxication [6,7].

Patients affected by hypothermia are frequently seen in the Emergency Department (ED). In the United States there are more than 1500 deaths per year reported as a result of accidental hypothermia in ED patients [8,9].

Our goal was to determine if there are factors associated with severity of illness by analyzing AH patients presenting to the ED.

## 2 Methods

We performed a cohort multi-institutional retrospective chart review of ED patients with a diagnosis of hypothermia, over a 40-month study period (January 1, 2012–April 30, 2015). These ED included two American College of Surgeons-verified Level 1 Trauma Centers, several tertiary academic medical centers, and suburban and urban hospitals, all located in the Northeast of United States. After Institutional Review Board (IRB) approval, subjects were identified using the International Classification of Diseases 9 (ICD-9) with codes for hypothermia, frostbite and drowning as the search criteria. We included only patients with primary (accidental) hypothermia. We excluded patients with initial rectal temperature >35 °C and patients with secondary hypothermia. Patient data included: age, gender, mechanism of injury, vital signs, and patient disposition. We used hospital admission as marker of illness severity. We examined the following factors: age, initial rectal temperature, found indoor, gender, blood alcohol level (BAL), and trauma. Student's t-test was used for differences between mean age and temperature for admitted and discharged patients. We calculated the percent of patients admitted by factor, the difference from overall admission rate and 95% confidence interval (CI) of difference.

The authors registered this research study in Research Registry under number 5361. This work has been followed the 2019 STROCSS (Strengthening the reporting of cohort studies in surgery) guidelines [10].

## 3 Results

Over the 40-month study period, 2094 patients were identified with a diagnosis of hypothermia. Of these, 132 patients with temperature ≤35 °C met the inclusion criteria. Table 1 shows ICD-9 codes used as the search criteria. Characteristics of AH ED patients are listed on Table 2.

**Table 1:** ICD-9 codes used as the search criteria.

Descriptors	ICD-9 code
Hypothermia-Associated with low environmental temperature	991.6
Frostbite	991.3
Drowning	994.1
Hypothermia-Not associated with low environmental temperature	780.65–780.99
Hypothermia-Newborn	778.3
Hypothermia-Anesthetic	995.89

**Table 2:** Characteristics of hypothermic ED patients.

Variable	Overall group (n = 132)
Mean age	55 ± 23 years
Female (%)	56 (42)
Mean temperature	32.8 ± 2.4 °C
Acute alcohol intoxication (%)	61/132 (46)
Mean blood alcohol level	230 ± 128 mg/dL
Positive toxicology: n (%)	21/61 (34)
Chronic alcohol abuse: n (%)	36/132 (27)
Causes of AH: n (%)	
• Outdoor exposure	87 (66)
• Found indoors	26 (20)
• Trauma	12 (9)
• Immersion	7 (5)
Seasonality: n (%)	
• Dec-Jan-Feb	58 (44)
• Mar-Apr-May	32 (24)
• Jun-Jul-Aug	14 (11)
• Sep-Oct-Nov	28 (21)
Severity: n (%)	
• Mild [32–34.9 °C]	108 (82)
• Moderate [28–31.9 °C]	21 (16)
• Severe [<28 °C]	3 (2)

For admitted and discharged patients the mean ages were 60 and 48 years, respectively (p = 0.01), and initial mean temperature 32.3 °C vs. 33 °C, respectively (p = 0.07). Found indoors was associated with an 86% admission rate, a 22% increase (95% CI, 3%–34%) compared to overall admission rate. However, the mean age of those found indoors was 75 ± 20 years, whereas those patients exposed outdoors were 49 ± 20 years. There was no statistically significant difference in admission rates from overall admission rate based on gender, alcohol or trauma. One patient died in the ED.

## 4 Discussion

We found that factors associated with increased severity of illness for hypothermic ED patients were older age and found indoors. Others have examined age and severity of illness. Hypothermia is the reported cause of death in approximately 1500 people per year in the United States, with roughly 50% occurring in individuals aged 65 and older [8,11]. In our study we found that patients admitted were older than those who were discharged. Elderly living in social isolation and with age-related mental decline have been shown to have increased vulnerability [12,13]. These factors combined with inefficiencies of the thermoregulation system associated with the aging may produce a situation in which hypothermia risk is greatly increased, even when indoors [14,15]. Indoor hypothermia has been reported in several studies and the associated outcome is suggested to be worse for vulnerable groups such as the elderly. It is thought that indoor hypothermia victims can fare worse than outdoor victims for the following reasons: they are not likely to be found as quickly, there is exposure to moderately cold temperatures for a longer period of time, the victims are more likely to be lightly clothed, and many of these indoor victims are found lying on the ground, which can promote cooling [14,16]. In our study 26 (20%) patients were found indoors, and of note, the only patient who died in the ED with AH was found indoor.

Based on our study, we conclude that for hypothermic ED patients increased severity of illness was associated with older age and found indoors but not associated with initial rectal temperature, gender, alcohol or trauma.

## 5 Limitations

Our study has a number of limitations. We performed a retrospective chart review which has innate problems [17]. Data abstracted can be different for different investigators. However, we dealt with differences in abstraction by having three investigators gathered the data from each chart and then resolved any differences by consensus. In general, we found good agreement between chart reviewers. The abstractors were blinded to the hypothesis of the paper and there were well defined objective data that were present in most of the charts as most of them were templated. The hypothermic patients were identified by ICD-9 codes associated with the ED physician diagnosis. As a result, some patients may have been given other diagnosis, such as head trauma, sepsis, etc. This may be have led to undercounting hypothermic patients. We do not believe that this affected the results of this study. Our study included 24 hospitals in Northeast United States. Results found in other areas may differ. Because this review was performed on ED patients, we do not have knowledge of complications and long term follow up. Our database does not include patients’ outcomes such as admission to intensive care unit and physiological markers and clinical indexes (APACHE II, SOFA, etc.). Patients' information including details on past medical history and medications.

## Provenance and peer review

Not commissioned, externally peer reviewed.

## Ethical approval

This study is a retrospective chart review, and as such, there is no need for an informed consent. The Atlantic Health System Institutional Review Board approved the study design and methodology.

## Sources of funding

None.

## Research registration Unique Identifying number (UIN)

Name of the registry: Research Registry.

Unique Identifying number or registration ID: researchregistry5361.

Hyperlink to your specific registration (must be publicly accessible and will be checked): https://www.researchregistry.com/browse-the-registry#home/registrationdetails/5e46ae6b15cac60015e088be/

## Guarantor

Patrizio Petrone, MD, PhD, MPH, MHSA, FACS.

## CRediT authorship contribution statement

**Patrizio Petrone:** Conceptualization, Data curation, Investigation, Methodology, Supervision, Writing - original draft, Writing - review & editing. **Corrado P. Marini:** Formal analysis, Investigation, Validation, Writing - original draft, Writing - review & editing. **Ivan Miller:** Conceptualization. **Collin E.M. Brathwaite:** Writing - review & editing. **Raelina S. Howell:** Writing - original draft. **Dennis Cochrane:** Conceptualization. **Wilson Rodríguez-Velandia:** Data curation. **Candela Rahn:** Data curation. **John R. Allegra:** Formal analysis, Investigation, Methodology, Validation, Writing - original draft, Writing - review & editing.

## Declaration of competing interest

None.
